# ST2 and regulatory T cells in the colorectal adenoma/carcinoma microenvironment: implications for diseases progression and prognosis

**DOI:** 10.1038/s41598-020-62502-0

**Published:** 2020-04-03

**Authors:** Guanglin Cui, Aping Yuan, Zhenfeng Li, Rasmus Goll, Jon Florholmen

**Affiliations:** 1 Department of Gastroenterology, the Second Affiliated Hospital of Zhengzhou University, Henan, China; 2 Faculty of Health Science, Nord University at Campus Levanger, Levanger, Norway; 3 Department of Gastroenterology & Nutrition, University Hospital of North Norway, Tromsø, University of Tromsø, Tromsø, Norway

**Keywords:** Gastroenterology, Colorectal cancer

## Abstract

ST2 (also known as IL1RL1) is the critical functional receptor for interleukin (IL)-33 in stimulating regulatory T cell (Treg) expansion and function in physiological and pathological conditions. We examined the correlation between ST2 cell expression and FoxP3 positive Tregs in both colorectal adenoma and cancer (CRC) microenvironment by real-time PCR, immunohistochemistry (IHC) and double immunofluorescences. The clinicopathological and prognostic significance of cellular ST2-positive cells and FoxP3-positive Tregs in patients with adenoma and CRC were evaluated. Real-time PCR results revealed increased expression levels of ST2 and FoxP3 mRNAs in both adenoma and CRC tissues as compared with control tissues. IHC analysis confirmed increased densities of ST2-positive cells in both the adenoma/CRC epithelium and stroma, which show a close positive linear association with the densities of FoxP3-positive Tregs in respective compartments. Pathological feature analysis showed that densities of ST2-positive cells in the tumor stroma were notably associated with degree of dysplastic grading in patients with adenoma, and disease stages and lymph node metastasis in patients with CRC. Kaplan-Meier survival curves suggested that CRC patients with high densities of ST2-positive cells in the stroma tend to have a shorter overall survival. We therefore concluded that increased densities of ST2-postive cells relate to Treg accumulation within the adenoma/CRC microenvironment, suggesting the IL-33/ST2 pathway as a potential contributor for immunosuppressive milieu formation that impact disease stage and prognosis in patients with CRC.

## Introduction

Colorectal cancer (CRC) is one of the most common malignant diseases worldwide. The progression of CRC is not only determined by CRC cells themselves but also by their immune microenvironment^1^. Although high densities of immune cells have been observed in the CRC microenvironment, most CRCs still progress invasively with the development of metastasis. Most likely, these patients do not develop a satisfactory antitumor immune capacity and some CRC cells have escaped the immune surveillance control^2^. Indeed, current scientific data strongly suggest that an immunosuppressive network is established in the CRC microenvironment wherein immune suppressive cells and cytokines play a critical role in inhibiting the host effective antitumor function, thereby promoting CRC progression and metastasis^3–5^. Therefore, there is a great interest in studying the contributing factors of immunosuppressive microenvironment formation during CRC development^6,7^.

Interleukin (IL)-33, which is a member of the IL-1 cytokine family, regulates a Th2 response but also a Th1 response through natural killer cells, CD8 T cells and γδ T cells^8–11^ and contributes to the generation of immunosuppressive cells^12–15^. Current evidence regarding the role of IL-33 in the CRC development suggest that IL-33 is involved in the pathogenesis of CRC^1,16–18^, and IL-33 significantly promotes the establishment and progression of CRC in both mice and humans^1,18–22^. Mechanisms whereby IL-33 promotes the development of CRC remain unclear, though several potential mechanisms have been hypothesized and evaluated^18,23–25^. Mertz KD *et al*. revealed that the promoting effect of IL-33 on the development of CRC in mice does not directly affect the proliferation of tumor cells in animal CRC models^20^. Other studies have demonstrated that IL-33 can greatly influence various immune cells including regulatory T cell (Treg) during differentiation, immune responses and homeostasis^10,26^. Thus, one of the proposed mechanisms is through the regulation of Treg expansion and function within the tumor microenvironment and by creating an pro-tumor microenvironment and diminishing innate anti-tumor immunity^15,17,26–29^. CD4+ and CD25+ Tregs, which are characterized by the expression of the master regulatory transcription factor forkhead box P3 (FoxP3), represent a subset of CD4+ T cells with a strong immunosuppressive capacity. In recent mice and human studies, an important role for Tregs in the development and progression of CRC has been demonstrated^30–36^. Extensive data have shown that the density of Tregs is significantly increased in the CRC microenvironment^30,31,34,35,37–39^, and elevated proportions of Tregs may have prognostic significance in patients with CRC^33,40–43^. More recently, a study performed in APC Min/+ mice examined the expression of Tregs in premalignant adenomatous lesions^44^. The results revealed that a high density of Tregs in the stroma was closely associated with the development of adenomatous polyps in the intestine^44^. We recently reported that the increase of FoxP3-positive Tregs is accompanied by the increased expression of the immunosuppressive cytokine IL-10 along the human colorectal adenoma-carcinoma sequence^44^. These findings suggest an association between Treg activation and the development and progression of CRC. Thus, there is currently a great interest in studying the regulatory mechanisms of Treg generation and function within the tumor microenvironment. Abundant evidence indicates that IL-33 is critically important for the generation and maintenance of Tregs in various colonic diseases^12,15,45–47^. Since ST2 (also known as IL-1RL1) is the critical functional receptor for IL-33 in regulating immune cell expansion and immune response, the importance of IL33/ST2 axis in the generation of immunosuppressive microenvironment in human tumors has got an intensively attention^18,48–50^. Current evidence have shown that a remarkable change in ST2 expression has been observed during the colorectal neoplastic transformation, which leads to the formation of adenoma^16^ and CRC^20^. In addition, we have previously demonstrated that several types of cells, including tumor cells, stromal cells and immune cells, express ST2 in human adenomas/CRCs^1^. Moreover, Mertz KD *et al*. reported that deficiency of ST2 could significantly protect the development of chemic carcinogen-triggered CRC in mice^20^. Another animal study showed that blocking ST2 signal by a specific antibody administration in the ApcMin/+ adenomatous polyps mice could remarkably inhibit the occurrence of polyps number and size compared to control mice^16^. Therefore, precisely study the significance of ST2 expression in the context of Treg biology might greatly help to understand IL-33’s role involved in the pathogenesis of CRC. Indeed, Pastille *et al*.^51^ have studied the important role of Tregs in the development of CRC in ST2fl/fl;Foxp3-Cre mice, with Treg-specific ST2 deletion, they have found that IL-33/ST2 axis plays a central role in shaping an immunosuppressive environment during intestinal tumorigenesis. Landskron G *et al*.^52^ investigated the potential role of IL-33/ST2 pathway in promoting metastasis of cancer cells by using CRC cell lines and CRC tissues. They reported that IL-33/ST2 axis in tumor microenvironment contributes to invasion and metastasis in left-sided CRC, most likely by the interaction between can associated fibroblasts and epithelial tumor cells and activating desmoplasia. Moreover, Li and colleagues^53^ analyzed IL-33 gene expression in human CRC tissues and carried out gene enrichment analysis with TCGA Data Portal, and studied CRC proliferation *in vivo* by inoculating MC38 tumors in IL-33 transgenic mice and investigated the cell proliferation *in vitro* with primary CRC cells isolated from fresh human CRC tissues, human CRC cell line HT-29 and mouse CRC cell line MC38. They concluded that IL-33 facilitates proliferation of colorectal cancer dependent on COX2/PGE2. Interestingly, Tregs are also reported to be a cellular source for COX-2^54,55^ and IL-33 could potentially stimulate Treg expansion and function via its functional receptor ST2^12,15,56^. These studies provided novel evidence to support the notion that Tregs contribute to the development of CRC. However, these results obtained from animal and cell line studies are waiting to be validated in human CRCs, particularly across the colorectal adenoma (premalignant lesion)-cancer sequence (malignant lesions).

Therefore, we designed this study to define dynamic relationship between ST2 and Tregs in different histological compartments across the colorectal adenoma-cancer sequence, evaluate its clinicopathological/prognostic significance in patients with adenomas and CRCs.

## Results

### The mRNA expression levels of ST2 and FoxP3 in adenoma and CRC tissues

**Figure 1:**
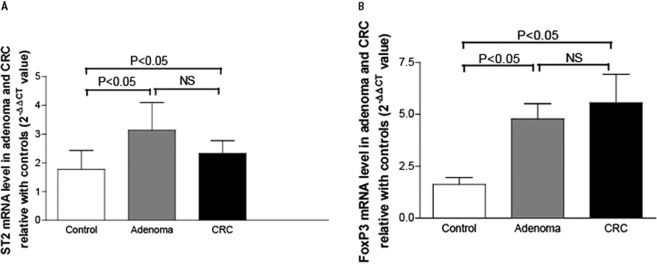
mRNA expression level of ST2 and FoxP3 in the adenomas and CRC tissues. The mRNA expression level of ST2 determined by real-time PCR was significantly increased in the adenoma tissues (*grey* bar in **A**) compared to the normal controls (*white* bar in **A**, *P* < 0.05). It was also significantly higher in the CRC tissues (*black* bar in **A**) than in the normal controls (*white* bar in **A**, *P* < 0.05) but slightly lower than that in the adenoma tissues (*grey* bar in **A**). As seen in **B**, the expression level of FoxP3 mRNA showed an increasing trend along the adenoma-carcinoma sequence. Increased expression of FoxP3 mRNA was started from the adenoma stage (*grey* bar in **B**) and even higher in the CRC stage (*black* bar in **B**) compared to the normal controls (*white* bar in **B**, both *P* < 0.05). NS: no significant, *P* > 0.05.

The quantitative PCR results are presented in Fig. 1. Compared with the controls, the mRNA expression of ST2 (Fig. 1A) were increased to a ~3-fold higher level in the adenoma tissues and ~2.3-fold higher level in the CRC tissues respectively (both *P* < 0.05). The expression level of ST2 mRNA was slightly higher in the adenoma tissues than that in the CRC tissues (*P* > 0.05).

Similarly, the mRNA expression of FoxP3 (Fig. 1B) were increased to a ~4.8-fold higher level in the adenoma tissues and to a 5.6-fold higher level in the CRC tissues (both *P* < 0.05).

### Cellular expression of ST2 and FoxP3 in the adenoma/CRC microenvironment

H&E stained images were supplied in Supplementary Data Fig. 1. IHC were used to examine cellular expression of ST2 and FoxP3 in the adenoma/CRC microenvironment.

**Figure 2:**
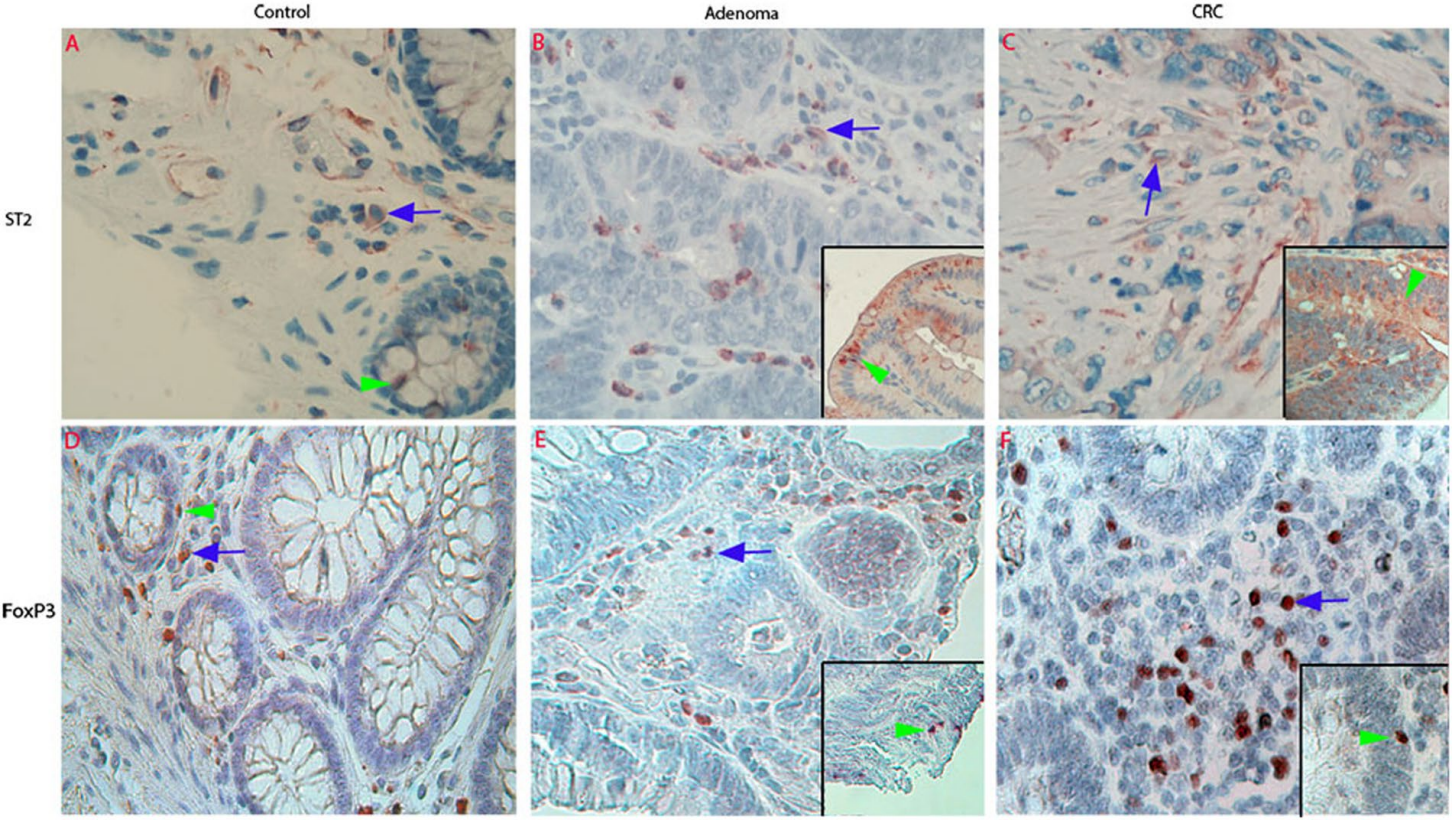
Photographic representations and density analysis of ST2-positive cells and Foxp3-positive Tregs in normal colorectal, adenoma and CRC tissues examined with immunohistochemistry (IHC). IHC images in first panel of Fig. 2 showed that ST2-immunoreactivity (IR) in the control tissues was observed in both the epithelium (*green arrowhead* in **A**) and lamina propria (*blue arrow* in **A**). In the adenoma and CRC tissues, increased ST2-IR was observed in both the adenoma (*green arrowhead* in inserted image in **B**)/CRC epithelium (*arrowhead* in inserted image in **C**) and adenoma (*blue arrow* in **B**)/CRC stroma (*blue arrow* in **C**). As seen in second panel of Fig. 2, low density of Foxp3-positive Tregs were observed in control lamina propria (*blue arrow* in **D**). However, dense Foxp3-positive Tregs were found in both the adenoma (*blue arrow* in **E**)/CRC stroma, (*blue arrow* in **F**), and some were infiltrated into adenoma/CRC epithelium (green *arrowhead* in inserted images in **E,F**). (**A–F**: IHC, counterstained with hematoxylin, original magnification 200×).

Following IHC staining, ST2-positive cells were observed in both the epithelium and lamina propria in normal controls (Fig. 2A), adenomas (Fig. 2B) and CRCs (Fig. 2C). Increased abundance of FoxP3-positive Tregs was also demonstrated in both the adenoma/CRC epithelium and stroma (Fig. 2E,F), compared with the controls (Fig. 2D).

**Figure 3:**
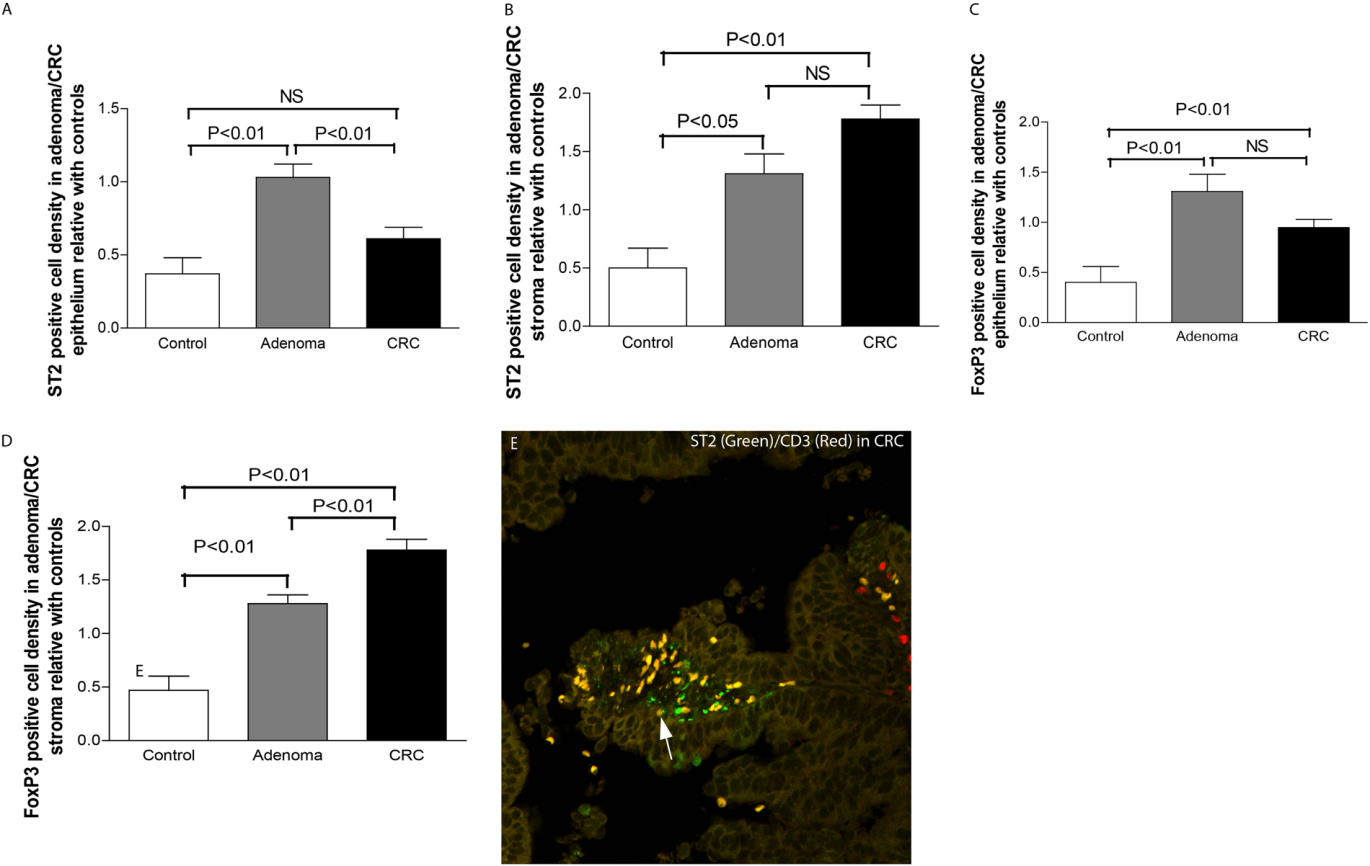
Density and phenotypical analysis of ST2-positive cells and FoxP3-positive Tregs in the adenoma/CRC stroma. Semiquantitative data showed that the scores of ST2-positive cells (**A,B**, epithelium and stroma respectively) and FoxP3-positive Tregs (**C,D**, epithelium and stroma respectively) in both the adenoma and CRC were increased. The density of ST2-positive cells and FoxP3-positive Trges were even higher in the adenoma epithelium (**A,C**, *grey* bar) and that in the CRC epithelium (**A,C**, *black* bar). Further phenotypic analysis of ST2-positive cells (*green* color in **E,F**) in the adenoma/CRC stroma defined that most positive cells were colocalized with CD3-positive labelled (*red* color in **E**) T lymphocytes (*white* arrow point in **E**) and some were SMA-alpha-labelled (*red* color in **F**) myofibroblasts (*white* arrow point in **F**). (**E**,**F**, confocal images, original magnification 200×; counterstaining was not applied).

The results of the semi-quantitative grading confirmed the above mentioned IHC results and revealed a trend of increasing densities of ST2-positive cells in the adenoma/CRC epithelium (Fig. 3A) and stroma (Fig. 3B). Notably, the density of ST2-positive cells in adenoma epithelium was higher than that in the control and CRC (see Fig. 3A, adenoma *vs*. control and adenoma *vs*. CRC, both *P* < 0.05). Increased densities of FoxP3-positive Tregs were also observed in both the adenoma/CRC epithelium (Fig. 3C) and stroma (Fig. 3D) compared with the controls. The density of FoxP3-positive Tregs was even higher in the CRC stroma than that in the adenoma stroma (Fig. 3D, *P* < 0.01).

Phenotypic analysis of ST2-positive cells in the adenoma/CRC stroma showed that many ST2-positive cells were CD3 positive lymphocytes (Fig. 3E).

To check whether FoxP3-positive cells infiltrated into adenoma/CRC epithelium were Tregs instead of epithelial cells, double immunohistochemistry with FoxP3/CD3 antibodies was performed. The image showed that FoxP3-positive cells in the adenoma/CRC epithelium were positive for CD3 (See Supplementary Data Fig. 2) and confirmed that the cells were lymphocytes, but not adenoma/CRC epithelial cells.

### Spearman’s R coefficient analysis of correlation between ST2 and FoxP3 at mRNA and cellular levels in the adenoma/CRC microenvironment

The analysis showed that the expression level of ST2 mRNA in both adenoma and CRC tissues were not correlated with the expression level of FoxP3 mRNA (see Table 1). However, ST2 expression at cellular level in the adenoma stroma was correlated with the density score of FoxP3-positive Tregs in the same compartment. The density of ST2-positive cells in both the CRC epithelium and stroma were correlated with the density scores of FoxP3-positive Tregs (Table 1).

**Table 1:** Spearman’s *R* coefficient analysis of correlation between ST2 and FoxP3 at mRNA and cellular levels in the adenoma/CRC microenvironment.

	ST2 in adenoma			ST2 in CRC		
	Tissue mRNA	Cells in epithelium	Cells in stroma	Tissue mRNA	Cells in epithelium	Cells in stroma
FoxP3 in adenoma						
Tissue mRNA						
*r*	0.1226					
*P*	0.4967					
Cells in epithelium						
*r*		0.3565				
*P*		0.0577				
Cells in stroma						
*r*			0.3899			
*P*			0.0365			
FoxP3 in CRC						
Tissue mRNA						
*r*				0.3149		
*P*				0.0962		
Cells in epithelium						
*r*					0.4279	
*P*					0.0053	
Cells in stroma						
*r*						0.4553
*P*						0.0028

### Analysis of ST2-positive cell and FoxP3-positive Treg densities in the tumor microenvironment against clinicopathological variables in patients with adenoma or CRC

We previously found that cytokines and their receptors may be expressed by a variety of cellular phenotypes in the CRC microenvironment^1,4,5,57,58^. Therefore, we analyzed correlations between the densities of ST2-positive cells and FoxP3-positive Tregs in different compartments of the adenoma/CRC microenvironment and the clinicopathological variables in patients with adenoma/CRC.

**Figure 4:**
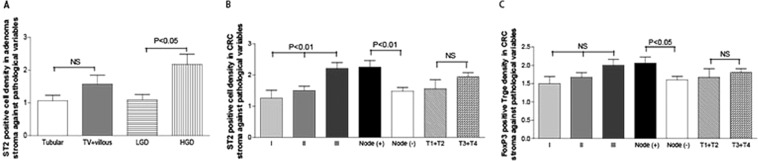
Clinicopathological analysis of ST2-positive cells and FoxP3-positive Tregs in patients with adenomas and CRCs. Further clinicopathological analysis revealed that increased densities of ST2-positive cells in the adenoma stroma (**A**) correlated with the degree of dysplasia, patients with high grade of dysplasia (HGD) tend to have a higher density of ST2-positive stromal cells than those with low grade of dysplasia (LGD). The density of ST2-positive cells in the CRC stroma correlates with TNM stages and node involvement (**B**), and the density of FoxP3-positive Tregs in the CRC stroma correlated with node involvement (**C**). NS: no significant, *P* > 0.05.

The results revealed that the density of ST2-positive cells in the adenoma stroma correlated with dysplastic degree grading, adenoma patients with high grading of dysplasia (HGD) tended to have a higher density of ST2-positive stromal cells than those with low grading of dysplasia (LGD) (Fig. 4A). Adenomas with villous feature has been shown to have a high risk to develop CRCs^59^. However, we could not find such correlation between adenomas with tubular type and those with villous features (Tubular vs. Tubularvillous+Villous: P > 0.05, see Fig. 4A). In addition, densities of ST2-positive adenoma epithelial cells correlated neither with histological types nor dysplastic degree grading (Supplementary Data Fig. 3A). In the CRC, the density of ST2-positive stroma cells was significantly associated with advanced TNM stage and node involvement (Fig. 4B). However, densities of ST2-positive cells in the CRC epithelium were not correlated with clinicopathological variables (Supplementary Data Fig. 3B).

The density of FoxP3-positive Tregs in both adenoma epithelium and stroma correlated neither with histological types nor dysplastic degree grading (Supplementary Data Fig. 3C,D). CRC patients with node involvement exhibited a higher density of FoxP3-positive Tregs in the tumor stroma compared to those without node involvement (Fig. 4C). However, densities of FoxP3-positive Tregs in the CRC epithelium were not correlated with clinicopathological variables (Supplementary Data Fig. 3E).

### Kaplan-Meier survival curves analyze the prognostic significance of ST2-positive cell and FoxP3-positive Treg densities in patients with CRC

**Figure 5:**
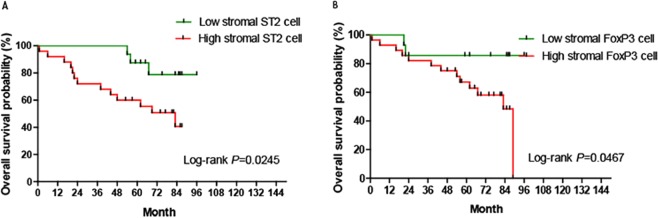
The Kaplan–Meier analysis of overall survival differences in CRC patients with differing density scores of ST2-positive cells and FoxP3-positive Tregs. The Kaplan–Meier analysis revealed that the density scores of ST2-positive cells (**A**) and FoxP3-positive Tregs (**B**) in the CRC stroma predicate the overall survival time in patients with CRC. CRC patients with high densities of ST2-positive cells and FoxP3-positive Tregs in the tumor stroma did have a significantly shorter overall survival time compared with those with low densities of ST2-positive cells and FoxP3-positive Tregs.

Kaplan–Meier analysis revealed that CRC patients with high densities of ST2-positive cells and FoxP3-positive Tregs in the tumor stroma tended to have a significantly shorter overall survival time compared with patients with low densities of ST2-positive cells and FoxP3-positive Tregs (Fig. 5A,B).

The densities of neither ST2-positive cells (Supplementary Data Fig. 4A) nor FoxP3-positive Tregs (Supplementary Data Fig. 4B) in the CRC epithelium were correlated with the survival rate of patients with CRC.

### Double immunohistochemistry images revealed an co-expression of ST2 with FoxP3

**Figure 6:**
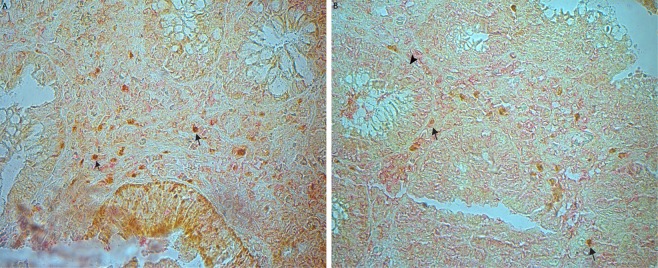
Photographic representations of ST2 expressed in FoxP3-positive Tregs in the adenoma stroma. Double immunohistochemistry images revealed that many FoxP3-labelled (*red* color in **A**) Tregs expressed ST2-IR (*brown* color in **B**) in a represent adenoma section. (**A,B**, double immunohistochemistry images, original magnification 400×; counterstaining was hematoxylin).

Double immunohistochemistry images revealed that many of T lymphocytes in the adenoma stroma (arrow pointed in Fig. 6A) and CRC stroma (arrow pointed in Fig. 6B) were positive for both FoxP3-IR (*brown* color) and ST2-IR (*red* color) in the representative sections. In addition, ST2-IR was also observed in the epithelial cells (arrowhead pointed in Fig. 6B).

## Discussion

The protumor effect of IL-33/ST2 pathway has been demonstrated in various types of cancers^1,19,20,60–63^. In this study, we examined expression of ST2 and its association with Treg accumulation in the adenoma/CRC microenvironment as well as the clinicopathological significance in patients with adenoma/CRC. Results showed that the expression of both ST2 and FoxP3 at the mRNA and cellular levels were significantly increased in the adenoma/CRC microenvironment. Moreover, the density of ST2-positive cells in the tumor stroma was correlated with the dysplastic degree in the adenoma, which is one of the important histological hallmarks for the progression of an adenoma towards to a CRC. Densities of ST2-positive cells in the CRC stroma were correlate with advanced TNM stage, node involvement and overall survival rate after surgery in patients with CRC. In addition, ST2 was frequently expressed in FoxP3-positive Tregs in the CRC microenvironment. These findings suggest that ST2 is involved in the regulatory effect of IL-33 on progression and prognosis of CRC.

Accumulating studies have demonstrated the importance of IL-33/ST2 pathway in regulating immune cell generation and function^12,15^. IL-33 can significantly enhance the recruitment of immunosuppressive immune cells into the tumor site, where these cells have a strong impact on immune microenvironment remodeling^15,17,29,51^. Exogenously administered recombinant mouse IL-33 significantly induces ST2-positive Tregs accumulated in tumor masses. In contrast, neutralizing IL-33 or ST2 by administration of antibodies remarkably decreases the density of ST2-positive Tregs inside tumor masses in CRC-bearing mice^63^. A more recent investigation has showed that the IL-33/Treg axis significantly contribute to the creating of tumor-promoting immune environment as seen in chronic inflammation condition^29^. Pastille E *et al*. further revealed that Tregs in the CRC microenvironment could preferentially upregulate ST2 expression in mice. Their transcriptomic and flow cytometry analyses demonstrate that ST2 expression induces a more activated and migratory phenotype in FoxP3 positive Tregs, and results in an intensively accumulation in the tumor microenvironment. Genetic ablation of ST2 could remarkably reduce Treg density, but enhance the density of effector CD8 positive T cells in the tumor microenvironment, thereby inhibiting CRC development in mice^51^. These data suggested that the IL-33/ST2 pathway plays an essential role in the regulation of Treg activation and function. In human CRCs, we and other group have shown that expression levels of IL-33 and ST2 are significantly increased^1,19^. Our present results demonstrated that increased expression of ST2 mRNA was increased in both adenoma and CRC tissues. Interestingly, the expression level of ST2 mRNA in the adenoma tissues was higher than that in the CRC tissues, which might imply that increased ST2 is an early event occurred in the adenoma stage of colorectal adenoma-carcinoma sequence and involved in the formation of adenoma. Our quantitative PCR data also showed that bot the expression of ST2 and FoxP3 mRNAs were increased in adenomas and CRCs respectively compared to the controls. Because ST2 is the primary functional receptor for IL-33, which contributes to Treg expansion and function^12,14,56^, our present results may imply that ST2 overexpression correlates to Treg accumulation in both the adenoma and CRC. IHC results confirmed that both densities of ST2-positive cells and FoxP3-positive Tregs were increased in the different compartments of adenoma/CRC microenvironment, and further double immunofluorescence images revealed that ST2 was frequently expressed in FoxP3-positive cells in the adenoma stroma. In addition, correlational analysis showed that cellular expression of ST2-positive stromal cells in the adenoma was correlated with the expression of FoxP3-positive Tregs in the adenoma stroma. Similar correlations between ST2-positive cells and FoxP3-positive Tregs in both the CRC epithelium and stroma were also found. These analysis may imply an regulation role of Tregs by IL-33 in during the establishment of CRC. Thus, one of likely explanations is that ST2 is a potential functional mediator for IL-33 in stimulating Treg accumulation in the adenoma/CRC microenvironment. Although FoxP3 is a commonly used biomarker for Tregs, however, we can still see that not all ST2 positive cells appear to be positive for FoxP3 in the Fig. 6. Indeed, a previous publication showed that FoxP3 is not exclusively expressed on bona fide CD4/CD25 positive Tregs and it is also expressed in CD44 positive effector and CD44/CD62L positive memory T cells upon stimulation^64^. Thus, we could postulate that ST2 might be also mediate the IL-33’s effect in other types of immune cells.

Moreover, previous studies have reported that IL-33/ST2 in human cancers is expressed in a variety of cells, including epithelial cells, stromal cells, infiltrating lymphocytes and Tregs, and microvessels^1,16,20,60,65^. In the CRC sections, our IHC results clearly demonstrated the increased expression of ST2-IR in the adenoma/CRC epithelium and tumor stroma, supporting the possibility that ST2 in the adenoma/CRC microenvironment is derived from a mixture of cell types.

With respect to the relationship between the density scores of ST2-positive cells and FoxP3-positive Tregs in different compartments and the clinicopathological features of patients with adenoma/CRC, we found that increased density of ST2-positive stromal cells was correlated with dysplastic degree grading in the adenoma, and with advanced TNM stages and lymph node involvement in the CRC. In addition, the increased density of FoxP3-positive Tregs in the CRC stroma was correlated with lymph node involvement. Taken together with previous findings of Tregs in suppressing host anti-tumor immunity^51,54^, our current finding may reflect the hypothesis that Tregs promote lymph node metastasis by limiting antitumor immunity and supporting tumor immune evasion. Current findings somewhat differ from our previous reports in which the mRNA expression levels of ST2 and FoxP3 mRNAs were not associated with clinicopathological features in patients with CRC^1,66^. This discrepancy may be explained by the fact that there are significant differences between the two techniques. Advantages and disadvantages of these two techniques have been discussed for years:^67–69^ we previously used quantitative real-time PCR as a high sensitive technique to quantify ST2 or FoxP3 at the mRNA level on only a tiny fraction of adenoma/CRC specimens. In current study, we used immunohistochemistry to precisely examine the cellular expression of ST2 and FoxP3 in different compartments of adenoma/CRC microenvironment on whole paraffin sections, as immunohistochemistry is an ideal technique to observe the location and semiquantitative expression of certain proteins in cellular and tissue levels. Therefore, combining these two techniques may be a better way to investigate the relationship between ST2 and Tregs within the tumor microenvironment in future studies.

Previous studies have also reported that IL-33 expression is correlated with survival in patients with various cancers^61,70,71^. We therefore performed a Kaplan–Meier analysis to investigate the relationship between the density scores of ST2-positive cells and FoxP3-positive Tregs located in different compartments (CRC epithelium/stroma) of the CRC microenvironment and the survival rate of patients with CRC. Our results revealed that densities of ST2-positive cell and FoxP3-positive Tregs in the CRC stroma, but not that in CRC epithelium, predicate the overall survival time after surgery. Since we have a small cohort of CRC patients with survival data in this study, it is necessary to extrapolate the conclusion in large scale samples in future studies.

**Figure 7:**
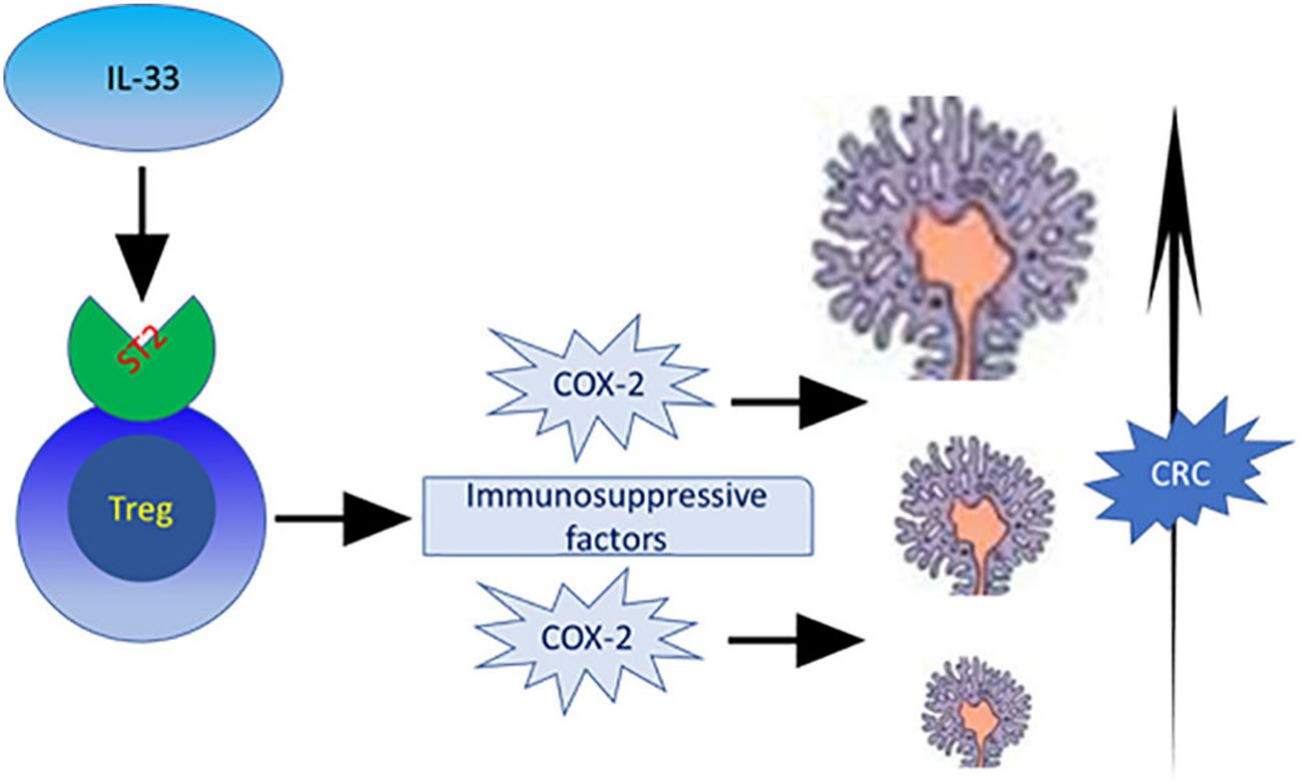
A schematic representation of the potential role of IL-33 functional receptor ST2 in contributing to the development of colorectal cancer.

Taken together with the results of previous studies, our current findings suggest that increased expression of ST2 in the tumor microenvironment, as a primary functional receptor for IL-33, was started from the adenoma stage and persisted to the CRC stage, which suggest that ST2 could be the functional receptor for IL-33 in promoting the development of adenoma/CRC (Fig. 7). The density of ST2-positive cells in the adenoma/CRC stroma parallels the increased density of FoxP3-positive Tregs in the adenoma/CRC microenvironment, and is associated with the some advanced clinicopathological variables in both the adenoma/CRC and prognosis in patients with CRC. Therefore, a future priority is to validate the current findings in a large cohort of patients with adenoma/CRC.

## Materials and methods

### Patients and biopsies

Fifty resected CRC specimens (male/female: 42/8; average age 42–89 years), 50 resected adenoma specimens (male/female: 31/19, ages 43–92 years) and 30 control colorectal tissues from health subjects with no pathological evidence by colonoscopic and microscopic examinations (male/female: 20/10; age 24–79 years) were included in this study. None of the included patients or control subjects had a history of regular use of immunomodulation treatment or chemotherapy. All biopsies were collected from the University Hospital of North Norway between August 2003 and December 2008, and detailed information on these adenomas and CRCs is summarized in Table 2. All biopsies were divided into two pieces. One piece was routinely embedded in paraffin, and sections were subsequently cut and stained with hematoxylin and eosin (*H&E*), another piece was embedded in *RNA later* solution for total RNA extraction and mRNA quantification. Routine histological diagnosis of all biopsies was performed in the Department of Clinical Pathology at the University Hospital of North Norway. The study protocol was approved by the Regional Ethical Committee of Northern Norway, and written informed consent was obtained from all included patients.

**Table 2:** Basic information of patients with adenoma and CRC.

	N	TNM			Invasion depth				Lymph node	
		I	II	III + IV	T1	T2	T3	T4	Positive	Negative
CRC	50	9	20	21	4	12	28	6	21	29
		Histological types			Degree of dysplasia					
		tubular	tubulovillous	villous	LGD		HGD			
Adenoma	50	33	15	2	40		10			

LGD: lower grade dysplasia; HGD: high grade dysplasia.

### Quantitative real-time PCR

The extraction of total RNA, reverse transcription of mRNA and real-time quantification of cDNA for the controls, adenomas and CRCs were performed using our previously published methods^1,72^. The primers for ST2 were purchased from Life Technologies (Cat# No. 4331182; Grand Island, NY, USA; which allows to amplify the membrane-bound but not the soluble ST2 isoform, as indicated by the manufacturer), FoxP3 and the housekeeping gene β-actin are also listed in our previous publications^1,66^ (see Table 3). The mRNA expression levels of ST2 and FoxP3 in colorectal adenomas and CRCs were measured according to the cycle threshold (CT) value relative to that of normal tissue. In brief, the expression levels of examined ST2 or FoxP3 mRNAs in the adenoma and CRC tissues were measured by CT cross-point value in relative to that of control tissues as fold difference (N) = 2^−ΔΔCT^, ΔCT = CT_examined gene (ST2 or FoxP3)_ − CT_beta-actin,_ ΔΔCT = ΔCT_Adenoma or CRC_-average ΔCT _normal_ as described in our recent publication^1,5,57^.

**Table 3:** Primer/probe sequences of house-keeping gene, ST2 and FoxP3 for quantitative real-time PCR.

Assay		Primer	Sequence
β-actin	TaqMan	Forward	5′ TGCCGACAGGATGCAGAAG 3′
		Reverse	5′ GCCGATCCACACGGAGTACT 3′
		Probe	FAM 5′ AGATCAAGATCATTGCTCCTCCTGAGCGC 3′ TAMRA
FoxP3-977	TaqMan	Forward	5′ GAGAAGCTGAGTGCCATGCA 3′
		Reverse	5′ GGAGCCCTTGTCGGATGAT 3′
		Probe	FAM 5′AATGGCACTGACCAAGGCTTCATCTGTG 3′ BHQ
ST2	TaqMan	Forward	purchased from Life Technologies (Cat# No. 4331182)
		Reverse	purchased from Life Technologies (Cat# No. 4331182)
		Probe	purchased from Life Technologies (Cat# No. 4331182)

### Immunohistochemistry (IHC)

To examine the expression pattern of ST2-positive cells and FoxP3-positive Tregs in the adenoma/CRC microenvironment, IHC staining was performed on 4-μm paraffin-embedded sections from controls, adenomas and CRC using the Vectastatin *Elite* ABC Kit (Vector Laboratories Inc, Burlingame, CA, USA) according to the manufacturer’s instructions and our previously published methods^4,5^. For epitope retrieval, deparaffinized and rehydrated slides were placed in a plastic jar with a boiling solution of 1 mM ethylenediaminetetraacetic acid (EDTA) (pH 8.0) for 20 minutes. The following primary antibodies were used: rabbit anti-ST2 polyclonal antibody (working dilution 1:100; Thermo Fisher Scientific, USA) and mouse anti-FoxP3 monoclonal antibody (working dilution 1:100, Abcam, UK). Tissue sections were incubated with the primary antibodies at 4 °C overnight. Then, the chromogen 3-amino-9-ethylcarbazole (AEC; Vector Laboratories, Burlingame, CA, USA) was used to detect the target antigens, and the tissues were counterstained with Mayer’s hematoxylin. Previous known tumor sections positive for ST2 and FoxP3 were used as positive controls to confirm the immunoreactivity of ST2 and FoxP3 in each series of IHCs. To exclude background staining by nonspecific antibody binding, negative controls were included using isotype-matched antibodies in each IHC test.

### Double immunohistochemistry

To check whether FoxP3-positive cells infiltrated into adenoma/CRC epithelium were Tregs instead of epithelial cells, double immunohistochemistry with FoxP3/CD3 (Rabbit polyclonal antibody, DAKO, Carpinteria, CA, USA) antibodies was performed with an EnVision G|2 doublestain dystem, rabbit/mouse kit (DAKO, Carpinteria, CA, USA) according to the protocol described in our previous publication^73^. In addition, the expression of ST2 in the FoxP3 positive Tregs with ST2/FoxP3 antibodies was examined with the same method. FoxP3-IR was developed with DAB substrate (*brown* color), and CD3-IR and ST2-IR were with permanent red substrate (*red* color). Since FoxP3 is positive in cell nuclear, nuclear counterstain with hematoxylin was not applied.

### Double immunofluorescence (DIF)

To examine the expression of ST2 on Treg cells in the adenoma/CRC microenvironment, DIF was performed with antibodies against ST2/FoxP3 in the selected sections of 10 normal controls, 10 adenomas and 10 CRCs according to the protocol described in our previous publications^1,5^. The immunoreactivity (IR) of FoxP3 was developed with a Texas red-conjugated secondary antibody, and ST2-IR was developed with a fluorescein isothiocyanate (FITC)-conjugated secondary antibody (both antibodies obtained from Jackson ImmunoRearch Lab., West Grove, PA, USA). Nuclear counterstaining was not applied.

To define the phenotypes of ST2-positive cells in adenoma/CRC stroma, DIFs with ST2/CD3 (Mouse monoclonal antibody, DAKO, Carpinteria, CA, USA) antibodies were performed according to the protocol described in our previous publications^1,5^. CD3-IR were developed with a Cy3-conjugated, and ST2-IR was developed with a fluorescein isothiocyanate (FITC)-conjugated secondary antibodies.

Negative controls were routinely performed in each DIF: (1) sections with isotype-matched antibodies incubation were used; (2) secondary antibodies were substituted with phosphate buffered saline (PBS) in each double immunofluorescence test.

### Morphometric analysis of IHC sections

Since we have observed that ST2-positive cells and FoxP3-positive Tregs were found in both the lamina propria and epithelium, we therefore evaluated their numbers in the lamina propria and epithelium according to the method described in our previous publications^1,66^. ST2-positive cells and FoxP3-positive Tregs in the adenoma/CRC stroma can be clearly viewed and counted, therefore, densities of ST2-positive cells or FoxP3-positive Tregs in the stroma, were semi-quantitatively scored in at least 3 high-power fields (HPF, ×400) with abundant distribution from each slide as follows: nil (0), 1–19 cells/field (1+), 20–49 cells/field (2+) and over 50 cells/field (3+). Whereas ST2-positive cells in normal/adenoma/CRC epithelial cell are diffusely stained and difficultly counted, ST2-positive cells and FoxP3-positive Tregs in the epithelium were graded on a scale of 0–3, with 0 representing no detectable staining and 3+ representing the strongest staining. Densities of ST2-positive cells or FoxP3-positive Tregs were scored by a histological researcher and a pathologist in a blinded manner respectively, then the average data were used as statistical analysis.

### Statistical analysis

The results were expressed as mean ± SEM (standard error of the mean) unless otherwise stated. Statistical significance was evaluated by the Mann–Whitney test or the Kruskal–Wallis test where appropriate. The correlation between the density of ST2-positive cells and the density of FoxP3-positive Tregs within the CRC microenvironment was analyzed using the nonparametric correlation Spearman’s R coefficient analysis. CRC patients with different densities of ST2- or FoxP3-positive cells were divided into “high or “low” groups according to the median value of positive cell densities, Kaplan–Meier analysis was used to calculate survival rates and differences in survival curves were determined by the log-rank test. Values of *P* < 0.05 were considered significant.

### Ethical approval and informed consent

**1. Approval:** Ethical approval was obtained by the Ethics Committee of University Hospital of North Norway.

**2. Accordance:** All procedures in this study were conducted in accordance with the Ethics Committee of University Hospital of North Norway.

**3. Informed consent:** Written informed consent was obtained from the patients.

## Supplementary information

Supplmentary information

## Data Availability

Data to support this study were based on analysis of immunohistochemistry and histological diagnosis from patients with adenoma and CRC admitted to our hospital.
