# Supplementary material for: ST2 and regulatory T cells in the colorectal adenoma/carcinoma microenvironment: implications for diseases progression and prognosis
Source: Sci Rep. 2020 Apr 3;10:5892. doi: 10.1038/s41598-020-62502-0 (PMC7125220; doi:10.1038/s41598-020-62502-0)
Supplement: Supplementary file 1 — Supplmentary information [file 41598_2020_62502_MOESM1_ESM.pdf]

Mar. 10, 2020

ST2 and regulatory T cells in the colorectal adenoma/carcinoma microenvironment: implications for diseases progression and prognosis

Guanglin Cui[1, 2], Aping Yuan[1], Zhenfeng Li[1], Rasmus Goll[3], Jon Florholmen[3]

[1]Department of Gastroenterology, the Second Affiliated Hospital of Zhengzhou University, China; guanlin.cui@nord.no; aping_yuan@yahoo.com; chenfenglee@126.com
[2]Faculty of Health Science, Nord University at Campus Levanger, Norway; guanglin.cui@nord.no
[3]Department of Gastroenterology & Nutrition, University Hospital of North Norway, Tromsø, University of Tromsø, Norway; rasmus.goll@unn.no; jon.florholmen@unn.no

All authors have read and approved this manuscript

Correspondence to: Dr. Guanglin Cui, Research Group of Gastrointestinal Diseases, the Second Affiliated Hospital of Zhengzhou University, China or Faculty of Health Science, Nord University, Campus Levanger, Norway
E-Mail: guanglin.cui@nord.no
Telephone: +47 74022557

Supplementary data

Legends for Supplementary figure

Supplementary Fig. 1. Representative *H&E* stained images to show histology of control (Suppl. Fig. 1A), adenoma (Suppl. Fig. 1B) and CRC (Suppl. Fig. 1C).

Supplementary Fig. 2. Representative double immunohistochemical image in the CRC section showed that some T lymphocytes infiltrated into the CRC epithelium (arrow pointed in Suppl. Fig. 2) and in the stroma (arrowhead pointed in Suppl. Fig. 2) were positive for both FoxP3-IR (*brown* color) and CD3-IR (*red* color).

Supplementary Fig. 3. Densities of ST2-positive cells in the adenoma epithelium (Suppl. Fig. 3A) and the CRC epithelium (Suppl. Fig. 3B) were not associated with clinicopathological variables in patients with adenoma and CRC respectively. Densities of FoxP3-positive Tregs in neither the adenoma epithelium (Suppl. Fig. 3C) nor stroma (Suppl. Fig. 3D) were associated with the adenoma pathological variables. Furthermore, densities of FoxP3-positive Tregs in the CRC epithelium were not correlated with clinicopathological variables (Suppl. Fig. 3E),

Supplementary Fig. 4. Kaplan–Meier survival analysis revealed that densities of ST2 positive cells (Suppl. Fig. 4A) and FoxP3 positive cells (Suppl. Fig. 4B) in the CRC epithelium did not predicate the survival of CRC patients after surgery.

Supplementary data

Fig. 1

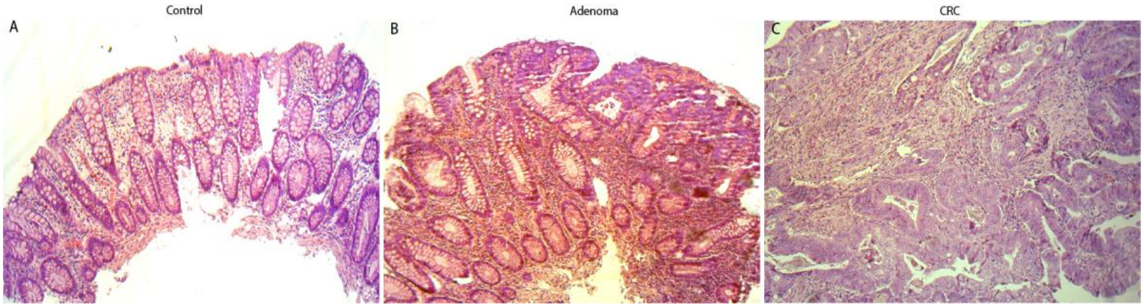

Fig. 2

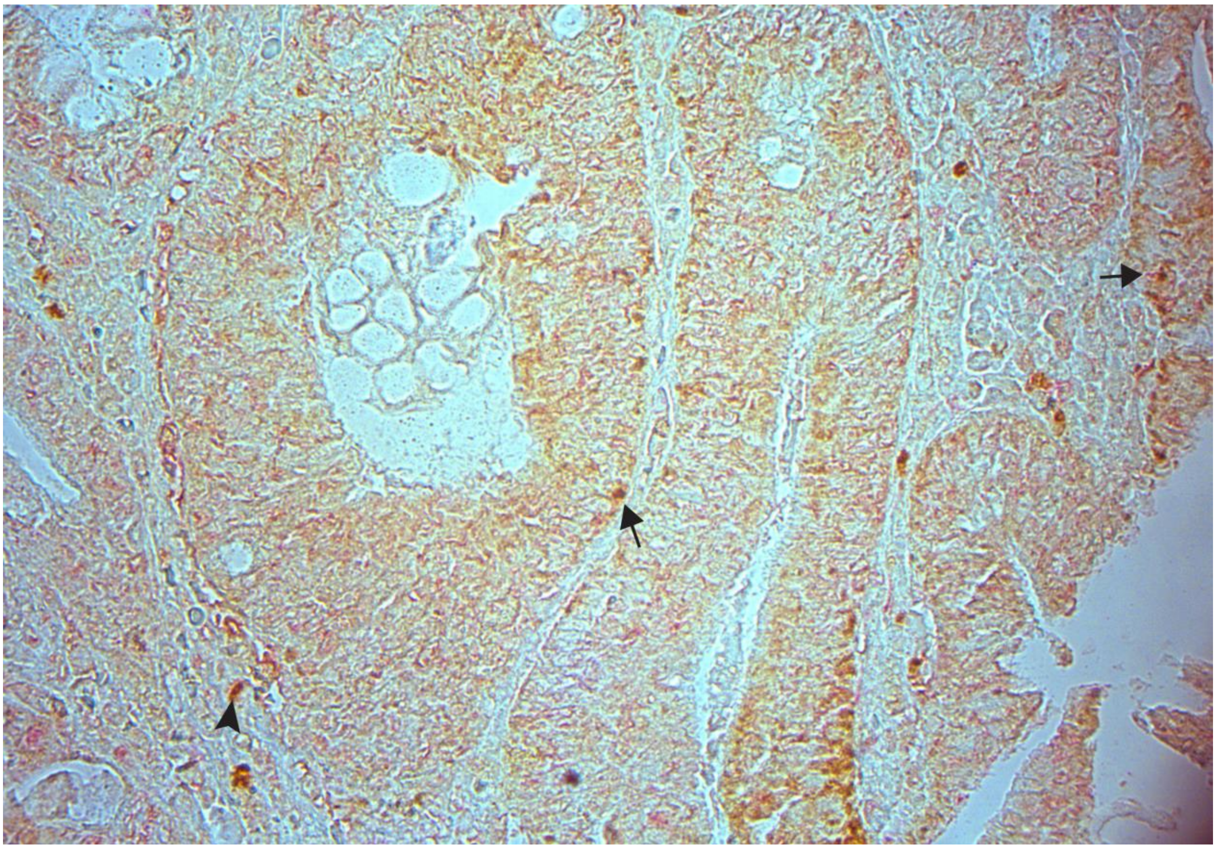

Fig. 3A

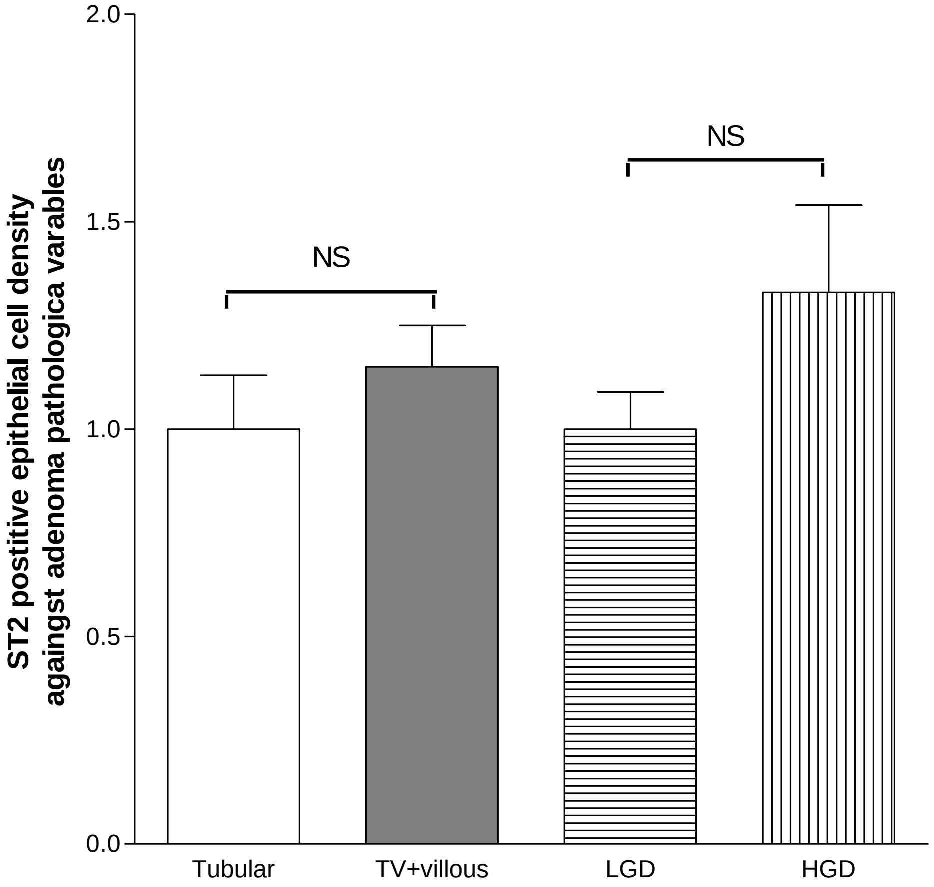

NS
NS
2.0
1.5
1.0
0.5
0.0
ST2 postitive epithelial cell density
againgst adenoma pathologica varables
Tubular
TV+villous
LGD
HGD

Fig. 3B

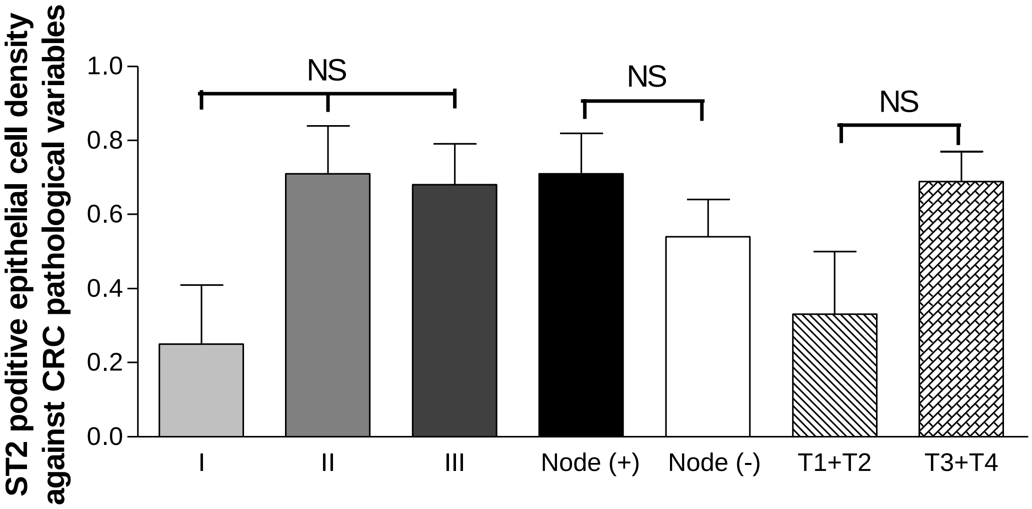
ST2 poditive epithelial cell density against CRC pathological variables
1.0
0.8
0.6
0.4
0.2
0.0
NS
NS
NS
I
II
III
Node (+)
Node (-)
T1+T2
T3+T4

Fig. 3C

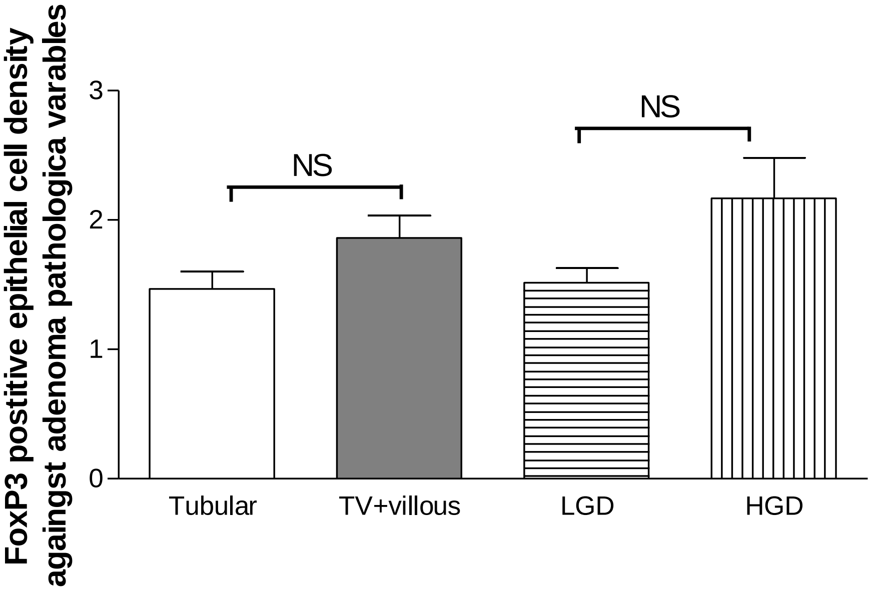
FoxP3 postitive epithelial cell density
againgst adenoma pathologica varables
0
1
2
3
NS
NS
Tubular
TV+villous
LGD
HGD

Fig. 3D

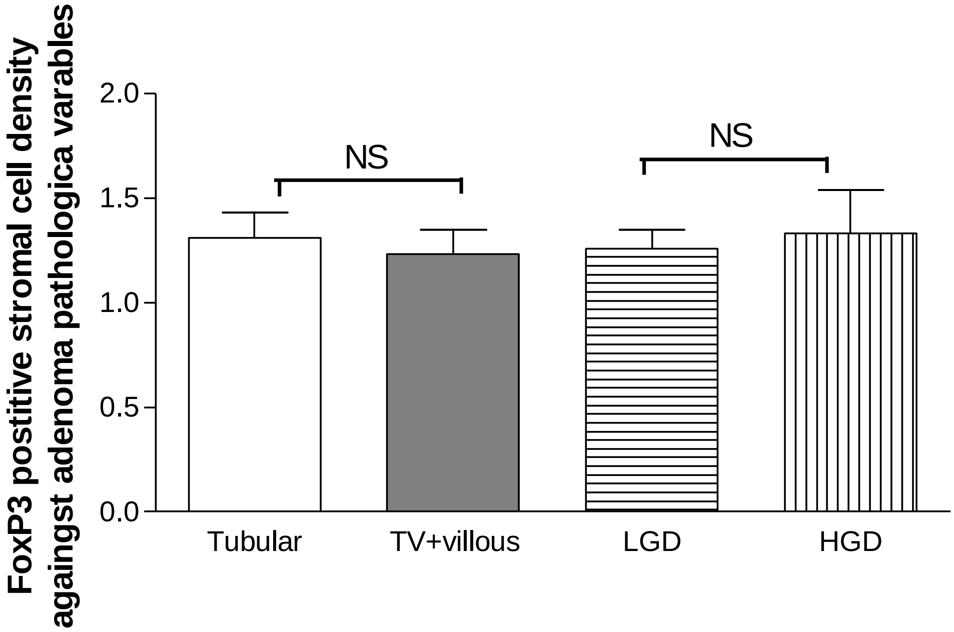

FoxP3 postitive stromal cell density
aagingst adenoma pathologica varables
2.0
1.5
1.0
0.5
0.0
NS
NS
Tubular
TV+villous
LGD
HGD

Fig. 3E

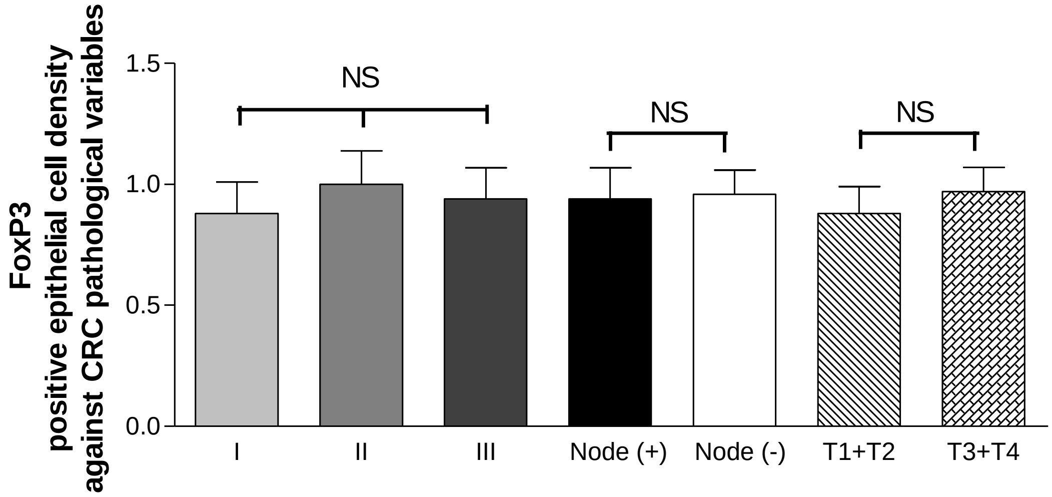

FoxP3
positive epithelial cell density
against CRC pathological variables
1.5
1.0
0.5
0.0
NS
NS
NS
I
II
III
Node (+)
Node (-)
T1+T2
T3+T4

Fig. 4A

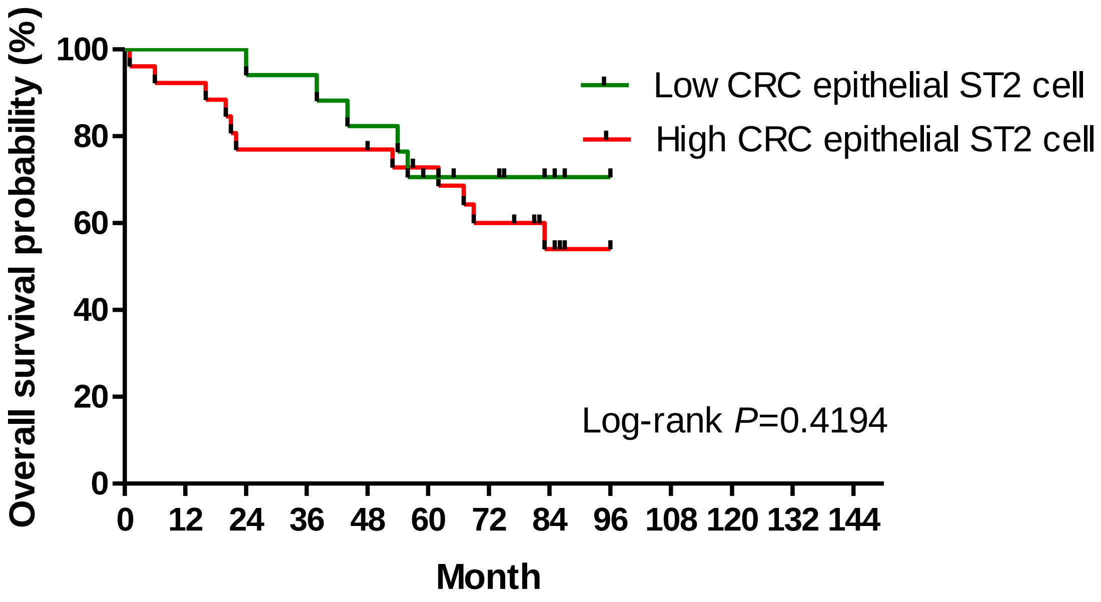

Overall survival probability (%)
100
80
60
40
20
0
0
12
24
36
48
60
72
84
96
108
120
132
144
Month
Low CRC epithelial ST2 cell
High CRC epithelial ST2 cell
Log-rank P=0.4194

Fig. 4B

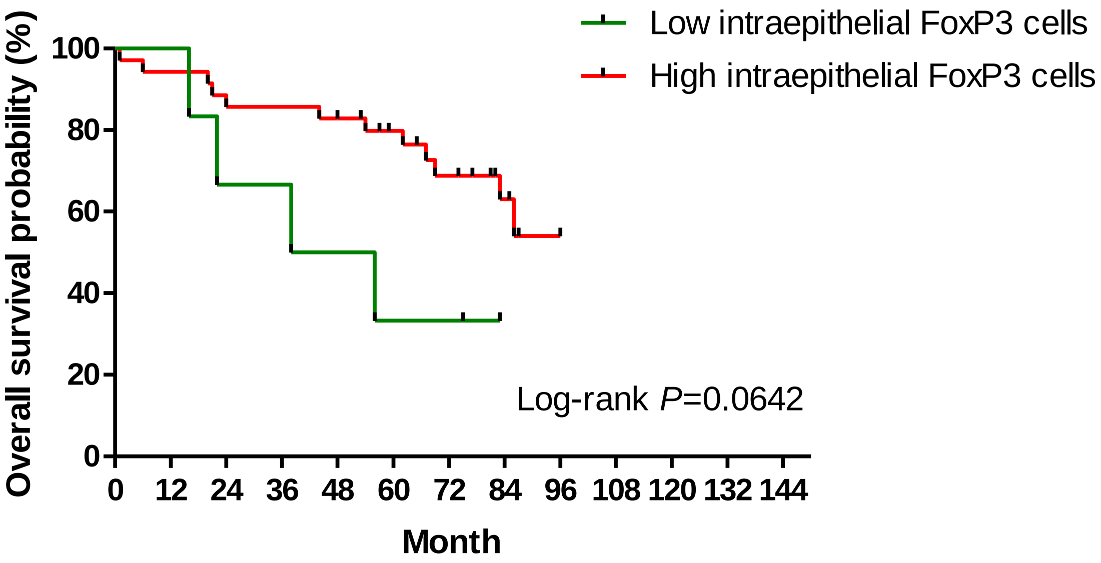

Low intraepithelial FoxP3 cells
High intraepithelial FoxP3 cells
Overall survival probability (%)
100
80
60
40
20
0
0
12
24
36
48
60
72
84
96
108
120
132
144
Month
Log-rank P=0.0642
